# Autophagy: A Novel Pharmacological Target in Diabetic Retinopathy

**DOI:** 10.3389/fphar.2021.695267

**Published:** 2021-06-21

**Authors:** Annagrazia Adornetto, Carlo Gesualdo, Maria Luisa Laganà, Maria Consiglia Trotta, Settimio Rossi, Rossella Russo

**Affiliations:** ^1^ Preclinical and Translational Pharmacology, Department of Pharmacy, Health and Nutritional Sciences, University of Calabria, Rende, Italy; ^2^ Multidisciplinary Department of Medical, Surgical and Dental Sciences, University of Campania “Luigi Vanvitelli”, Naples, Italy; ^3^ Department of Experimental Medicine, University of Campania “Luigi Vanvitelli”, Naples, Italy

**Keywords:** autophagy, diabetic retinopathy, LC3, autophagosomes, retinal degeneration, hyperglycemia

## Abstract

Autophagy is the major catabolic pathway involved in removing and recycling damaged macromolecules and organelles and several evidences suggest that dysfunctions of this pathway contribute to the onset and progression of central and peripheral neurodegenerative diseases. Diabetic retinopathy (DR) is a serious complication of diabetes mellitus representing the main preventable cause of acquired blindness worldwide. DR has traditionally been considered as a microvascular disease, however this concept has evolved and neurodegeneration and neuroinflammation have emerged as important determinants in the pathogenesis and evolution of the retinal pathology. Here we review the role of autophagy in experimental models of DR and explore the potential of this pathway as a target for alternative therapeutic approaches.

## Introduction

Diabetic retinopathy (DR), a chronic and progressive complication of diabetes mellitus, is the main cause of legal blindness in working-age population (20–65 years old) (Ting et al., 2016; Simo-Servat et al., 2019). DR is prompted by hyperglycemia, which causes an increase of oxidative stress leading to an adaptive inflammatory response in microvasculature and neuroretinal tissue (Saxena et al., 2016; Al-Kharashi, 2018). The disease has long been considered as a microvascular disease, since loss of pericytes, damage of vascular endothelial cells and breakdown of blood-retinal barrier (BRB) are typical hallmarks of the early stage of the pathology (Beltramo and Porta, 2013; Mrugacz et al., 2021). However, recent and intensive research identified neurodegeneration and neuroinflammation as processes involved in the pathogenesis and evolution of DR (Kadlubowska et al., 2016). Furthermore, experimental and clinical studies have shown that neuronal apoptosis and reactive gliosis, with thinning of the nerve fiber layer often precede the typical vascular alterations (Barber et al., 2011; Gu et al., 2019). Importantly, DME (diabetic macular edema), which is due to an abnormal intraretinal fluid collection in the macular area, is the most common cause of vision loss in patients with DR (Romero-Aroca et al., 2016). Experimental and clinical studies have highlighted the role of inflammation in DME and OCT-imaging biomarkers of retinal inflammation have been identified (Ceravolo et al., 2020).

The mechanisms underlying the neurodegenerative and neuroinflammatory processes occurring in DR are common to other central and retinal diseases, like glaucoma and retinitis pigmentosa (Baumgartner, 2000; Barber, 2003; Gupta and Yücel, 2007). These mechanisms include oxidative stress and free radical formation, advanced glycation end products (AGEs) production, glutamate excitotoxicity, mitochondrial dysfunction, impaired bioenergetics, dysfunction of neurotrophin signals and autophagy (Dong et al., 2009; Jellinger, 2010; Rosa et al., 2016).

Autophagy is a major lysosomal pathway for the turnover of cytoplasmic organelles and long-lived proteins and, besides its homeostatic functions, it also acts as an adaptive response to cellular stresses (Mizushima, 2007). Dysfunctions of this process have been identified as recurrent events in neurodegenerative disorders (Frake et al., 2015) and, more recently, experimental and clinical data have shown that autophagy modulation also occurs in experimental models of DR and in the retina of diabetic patients, with or without retinopathy (Lopes de Faria et al., 2016; Dehdashtian et al., 2018).

However, the functional role of autophagy in DR remains unclear. Here we discuss the available literature on the role of autophagy in experimental models of DR and explore the potential of this pathway as a target for alternative therapeutic approaches.

## Diabetic Retinopathy: A Neurodegenerative Retinal Disease

DR is a social disease with considerable costs, whose global incidence is strongly increasing due to the improved life expectancy and the exponential spread of diabetes (Flaxman et al., 2017). Indeed in 2015, 415 million people were affected by diabetes globally and this number is projected to reach 642 million by 2040 (Ogurtsova et al., 2017). In addition, it has been estimated that more than a third of people with diabetes worldwide have some form of DR and that nearly one in 10 develops forms of DR or complications that are particularly threatening for the sight such as proliferative DR or diabetic macular edema (Yau et al., 2012).

The diagnosis of DR is made on the bases of typical vascular abnormalities following the clinical examination of ocular fundus; it is possible to distinguish two stages: the non-proliferative DR (NPDR) and the proliferative DR (PDR). NPDR, the earliest form of DR, is divided into three stages of increasing severity, namely: 1) the mild DR characterized by rare microaneurysms and hemorrhages; 2) the moderate DR characterized by an increase in the aforementioned lesions associated with hard exudates; 3) the severe or pre-proliferating DR characterized by the coexistence of numerous microaneurysms, retinal hemorrhages, cottony nodules and venous caliber anomalies (Singh et al., 2008; Karst et al., 2018). At this stage, diabetic subjects can sometimes be asymptomatic for long time. On the other hand, in PDR, characterized by the appearance of epiretinal or epi-papillary new vessels that can sometimes invade the vitreous, patients may present a sudden vision impairment due to vitreous hemorrhages and/or tractional retinal detachment (Gotzaridis et al., 2001). Both forms of DR can be further complicated by macular edematous (DME) and/or ischemic damage, which are the main causes of severe vision impairment (Wilkinson et al., 2003; Cheung et al., 2010; Wang and Lo, 2018).

**FIGURE 1:**
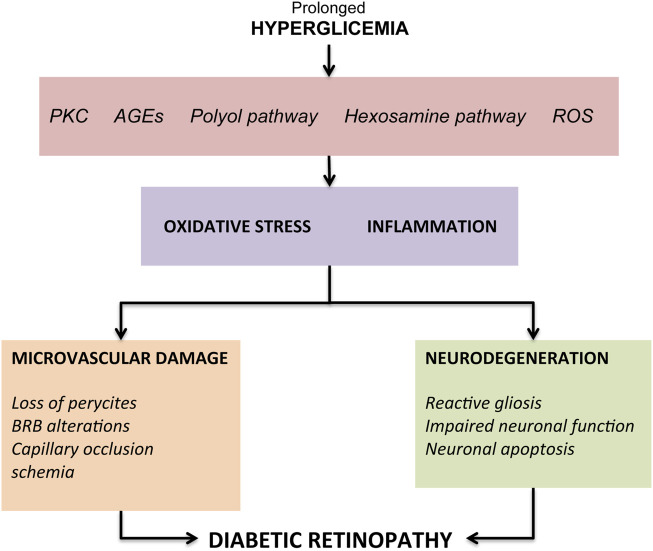
Overview of the pathogenic mechanisms leading to diabetic retinopathy.

The onset of typical DR vascular changes is determined by prolonged hyperglycemic episodes resulting from suboptimal glycemic control in patients with type I or II diabetes mellitus. Elevated blood glucose levels result in aberrant regulation of a number of biochemical pathways, eventually leading to superoxide production and overload of oxidative stress in retinal tissues. Prolonged hyperglycemia has been shown to cause increased flow of the polyol pathway (Gabbay et al., 1966; Lee and Chung, 1999), increased formation of AGEs (Shinohara et al., 1998; Stitt, 2010), abnormal activation of signaling cascades like protein kinase C (PKC) pathway (Koya and King, 1998; Idris et al., 2001), increase in the flux of the hexosamine pathway (Kolm-Litty et al., 1998; Du et al., 2000) and reactive oxygen species (ROS) (Brownlee, 2005). All these changes lead to an intensification in oxidative stress and an inflammatory attack on the retina with consequent structural and functional changes (Kowluru and Chan, 2007; Hammes, 2018) (Figure 1).

The first responses, considered as metabolic self-regulation to increase retinal metabolism, are vessel dilation and changes in blood flow (Bek, 2017). Another hallmark of early DR is the loss of pericytes which has been demonstrated in both *in vitro* and *in vivo* studies (Romeo et al., 2002). Since pericytes provide structural support to capillaries, their loss leads to the formation of microaneurysms, which represents the first clinical characteristic sign of DR (Ejaz et al., 2008). Other pathogenetic processes found during DR include endothelial cells apoptosis and thickening of the basement membrane, which overall contribute to compromise the BRB integrity (Beltramo and Porta, 2013). Finally, the loss of pericytes and endothelial cells causes capillaries occlusion and consequent ischemia. Retinal ischemia, through the activation of hypoxia-inducible factor-1 (HIF-1) (Huang et al., 2015), determines an overproduction of vascular endothelial growth factor (VEGF), a key factor involved in both the progression of retinopathy towards PDR, and in DME development through phosphorylation of tight junction proteins such as occludin and zonula occludens-1 (Antonetti et al., 1999). Furthermore, VEGF, by the activation of the mitogen activated protein (MAP), stimulates the proliferation of endothelial cells, resulting in new vessels development (Rousseau et al., 1997). The key role of VEGF in DR has been demonstrated in multiple studies showing its increased expression in diabetic mice retina (Li et al., 2010; Rossi et al., 2016), as well as in the vitreous of patients with DME and PDR (Adamis et al., 1994). In light of these evidence, the intravitreal injection of anti-VEGF agents is currently the gold standard for both early and advanced stages DR therapy (Sun and Jampol, 2019). Other therapeutic tools aimed at managing the microvascular complications of DR are steroid intravitreal injection, laser photocoagulation and vitreous surgery (Stitt et al., 2016; Wang and Lo, 2018; Sun and Jampol, 2019). However, although these treatments demonstrate clinical benefits, no tools are effective in completely blocking clinical progression or reversing retinal damage. In fact, such therapies are often used in the more advanced stages of DR, characterized by a high risk of irreversible and severe visual impairment.

Furthermore, in many cases frequent administration of intravitreal agents is necessary with a consequent increased risk of side effects related to the injection, not to mention the high costs associated with frequent eye examinations (Donnelly et al., 2004; Gupta et al., 2013).

For many years, microangiopathic lesions were considered the exclusive cause of DR, leading to the visual loss in diabetic patients. However, the concept of DR as a microvascular disease has evolved: nowadays, it is considered a more complex diabetic complication, in which neurodegeneration has emerged as an important factor, playing a significant role in DR pathogenesis and evolution (Ola and Alhomida, 2014; Jindal, 2015). Indeed, the American Diabetes Association (ADA) recently defined DR as a diabetes neurovascular complication that involves a progressive disruption of the interdependence between multiple cell types in the retina (Solomon et al., 2017).

The hallmarks of diabetes-induced neuroglial degeneration, which include reactive gliosis, impaired retinal neuronal function and apoptosis of neural cells, have been described before typical microangiopathy in multiple experimental models of DR and also in the retina of diabetic donors (Barber et al., 1998; Lieth et al., 1998; Lung et al., 2012; Howell et al., 2013; Jindal, 2015).

The first retinal neurons affected are retinal ganglion cells (RGCs) and amacrine cells, however photoreceptors also show an increased apoptosis (Lynch and Abràmoff, 2017). The structural consequence of this early death is a reduction in the ganglion cell layer (GCL) thinning and corresponding loss of nerve fiber layer (NFL) thickness, detected by optical coherence tomography (OCT) (van Dijk et al., 2010; Sohn et al., 2016). Moreover, functional studies performed with multifocal electroretinography (mfERG) have shown a delayed implicit time P1 and a reduction in the trace’s amplitude as a consequence of the early neurodegenerative process (Simão et al., 2017). These structural and functional alterations lead to reduced contrast sensitivity, delayed dark adaptation and altered visual fields, resulting overall in reduced vision-related quality of life, despite the absence of clinically detectable vascular anomalies (Wolff et al., 2015; Trento et al., 2017).

Müller cells and retinal astrocytes play an important role in the damage to retinal neurons and in linking the neurodegenerative process with vascular disease. Indeed, gliosis is associated with higher expression of VEGF and hyper-activation of pro-inflammatory pathways, with consequent overexpression of pro-inflammatory cytokines and dysfunction of the BRB (Bringmann and Wiedemann, 2012). Diabetes-induced subclinical inflammation is further amplified by the activation of immune cells resident in the retina, namely microglial cells. This microglial activation is accompanied by a phenotypic shift from the anti-inflammatory (M2) towards a pro-inflammatory amoeboid (M1) form (Coorey et al., 2012; Arroba and Valverde Á, 2017). This shift results in transcriptional changes mediated by nuclear factor-kappa B (NF-κB) and extracellular signaling mechanisms of the signal-regulated kinase (ERK) responsible for the release or activation of pro-inflammatory and neurotoxic molecules (i.e. cytokines, chemokines, glutamate) which contributes to the disruption of BRB and neuronal death (Altmann and Schmidt, 2018).

## Autophagy: Mechanisms and Functions

Autophagy is a highly conserved catabolic pathway by which cells remove misfolded or aggregated proteins and damaged organelles (Klionsky et al., 2021). This process regulates essential biological functions such as cell survival, cell metabolism, development, aging, and immunity. It also represents an adaptive response to different forms of stresses, like nutrient deprivation, growth factor depletion, infection, hypoxia, ischemia/reperfusion injury, oxidative stress, endoplasmic reticulum (ER) stress and mitochondrial damage (Glick et al., 2010; Dikic and Elazar, 2018).

In mammalian cells, there are three primary types of autophagy: microautophagy, macroautophagy, and chaperone-mediated autophagy (CMA) (Yang and Klionsky, 2010). Furthermore, different selective forms of autophagy, such as mitophagy, ribophagy or aggrephagy, have also been identified (Menzies et al., 2017).

In microautophagy, cytosolic components are directly taken up by lysosomes through the invagination of their membrane (Li et al., 2012). CMA, involves the formation of a complex between target proteins (identified by bearing a CMA targeting motif) and chaperones of the Hsp70 family; these complexes are recognized by the lysosome-associated membrane protein type-2A (LAMP-2A) at the lysosomal membrane where the substrate proteins unfold and translocate in the lumen for degradation by lysosomal hydrolase (Itakura and Mizushima, 2010).

Macroautophagy (hereafter referred to as autophagy) involves the formation of a cup-shaped membrane structure, the phagophore, that elongates and closes around the cytosolic cargo; the resulted double-membrane vesicle is called autophagosome and it is selectively associated with this pathway (Yang and Klionsky, 2010). Autophagosomes are transported, along the microtubules, to the perinuclear region where they fuse with lysosomes; here the autophagic content is degraded and released for recycling into the cytoplasm (Parzych and Klionsky, 2014). Autophagy and its regulatory mechanisms are evolutionarily conserved among eukaryotic cells even if the level of complexity of the process may differ (Yang and Klionsky, 2009).

Autophagosome biogenesis is orchestrated by the sequential action of autophagy-related (Atg) proteins; most of them were originally identified in yeast but have their homologs in mammalian cells (Mizushima et al., 2011). The ULK1 complex, formed by the serine/threonine protein kinase Atg1/(unc-51-like kinase 1), FIP200 (focal adhesion kinase family interacting protein of 200 kDa), Atg13 and Atg101 is involved in the initiation of autophagy (Hara et al., 2008). Upon autophagy induction, the mammalian target of rapamycin (mTOR), one of the main negative regulators of the process, is inactivated resulting in upregulation of ULK1 kinase activity and consequent phosphorylation of Atg13 and FIP200 (Noda, 2017). ULK1 complex gathers to specific ER region engaged in autophagosome formation (Itakura and Mizushima, 2010) and regulates the recruitment of a second kinase complex, the vacuolar protein sorting 34 (Vps34) complex formed by Beclin-1, AMBRA, Vps34, Vps15 and Atg14 (Glick et al., 2010). Vps34 participates in various membrane-sorting processes but it is selectively involved in autophagy when complexed to Beclin-1 (Backer, 2008). At variance with the other PI3-kinases, Vps34 only uses phosphatidylinositol (PI) as substrate to generate phosphatidyl inositol triphosphate (PI3P), which is therefore essential for phagophore nucleation, elongation and recruitment of other Atg proteins (Xie and Klionsky, 2007). The interaction of Beclin-1 with Vps34 promotes its catalytic activity and increases PI3P levels (Glick et al., 2010).

Following the initiation step, the elongation process is undertaken by two ubiquitin like proteins: Atg12 and Atg8/LC3. In this system, the E1-like enzyme Atg7 and E2-like enzyme Atg10 catalyze the formation of the Atg12-Atg5 complex that allows the formation of the Atg12-Atg5-Atg16 (L1) complex. The latter is crucial for autophagosome formation and for efficient promotion of the microtubule-associated protein light chain 3 (LC3) lipidation (Otomo et al., 2013).

Several experimental evidences demonstrate that LC3 is involved in the selective identification of autophagy substrates (Yoshii and Mizushima, 2017; Mizushima and Murphy, 2020). Indeed, LC3-II interacts with the constitutively expressed adaptor molecule p62 (or sequestosome-1, SQSTM1) that contains both a ubiquitin binding domain and a LC3-interacting (LIR) domain to deliver sequestered proteins to the autophagosomes (Zhang et al., 2015).

The fusion of the autophagosomal membrane with lysosome results in the release of a single-membrane autophagic body into the lysosomal lumen, which is followed by the degradation of the autophagic cargo by the lysosomal acid proteases (Dikic and Elazar, 2018).

Cellular homeostasis depends on the balance between the production and removal of macromolecules and organelles. In this context, basal autophagy activity plays a key role in the maintenance of cellular integrity (Chun and Kim, 2018). As a quality control mechanism, the process is fundamental for every cell, but it is particularly important in neurons. Indeed, neuronal cells are metabolically highly active and, being post mitotic cells, cannot dilute damaged or aged organelles and misfolded proteins by cell division (Mariño et al., 2011; Russo et al., 2013). Therefore, not surprisingly, accumulation of these altered components, due to autophagy inefficiency, has been associated with neurotoxicity and neurodegeneration.

Autophagy disruption or insufficiency has been reported in a number of different ocular diseases and pathological conditions like: retinal injury (Besirli et al., 2011), retinal degenerations (Punzo et al., 2009; Rodríguez-Muela et al., 2015), light-induced stress (Kunchithapautham et al., 2011; Chen et al., 2014), hyperglycemia (Lopes de Faria et al., 2016) and hypoglycemia (Zhou et al., 2015).

In this context, autophagy is becoming an attractive target to treat neurodegenerative disorders (Zhu et al., 2013), including the ones affecting the retina (Russo et al., 2013).

## The Role of Autophagy in Animal Models of Diabetic Retinopathy

Several groups have reported a modulation of Atg proteins in animal models of TD1 and TD2 diabetes (Table 1). In C57BL/6J mice, induction of type1 (T1D) diabetes by administration of streptozotocin (STZ) was associated with increased LC3-II immunoreactivity in the outer plexiform layer (OPL) and upregulation of the Atg related proteins, Beclin-1 and Atg5 (Piano et al., 2016). These changes occurred within the same time frame of outer retinal damage and might take part to the process of photoreceptors loss in the early phase of DR, before the appearance of evident signs of vascular damage (Piano et al., 2016). Accordingly, upregulation of Beclin-1, LC3-II, Atg12-Atg5 and Atg3 was reported in STZ-diabetic rats and Ins2^Akita^ mice, a spontaneous T1D mouse model (Wang et al., 2017). In this study knockdown of Hist1h1c, a gene that encoded for Histone H1.2 protein, significantly reduced both basal and high-glucose-induced autophagy, attenuated inflammation and cell toxicity. Conversely, adeno-associated virus (AAV)-mediated histone HIST1H1C overexpression led to increased autophagy, glial activation and neuronal loss which are pathological changes identified in the early stages of DR (Wang et al., 2017). These findings suggest that over-stimulation of autophagy is associated with increased retinal cell death and takes part to the progression of DR through advanced stages.

**TABLE 1:** Autophagy modulation in animal models of diabetic retinopathy.

Animal Models	DR model	Autophagy markers	References
Mice			
C57BL/6J	150 mg/kg STZ (single injection)	↑ LC3-I	Piano et al. (2016)
		↑ Atg5	
		↑ Beclin-1	
Ins2^+/+^ Akita (male)		↑ Beclin-1	Wang et al. (2017)
		↑ LC3-II	
		↑ Atg12-Atg5	
		↑ Atg3	
C57BL/6J (male, 6 weeks old)	40 mg/kg STZ (5 days treatment)	↓ Atg9	Qi et al. (2020)
		↓ Atg7	
		↓ LC3	
		↓ Beclin-1	
C57BL/KsJ-db/db (male, 8-12-16-18-25 weeks old) and -db/m (male, 8 weeks old)		Fluctuating modulation of LC3-II	Fu et al. (2020)
C57BL/6J (male, 6 weeks old)	50 mg/kg/d STZ	↓ Beclin-1	Wang et al. (2020)
		↓ Atg7	
		↓ p62	
		↓ LC3-II	
C57BL/6J (male, 8 weeks old)	150 mg/kg STZ (single injection)	↑ Beclin-1	Madrakhimov et al. (2021)
		↑ ATG9A	
db/db mice (male, 20 weeks old)		↓ LC3-II	Luo et al. (2021)
		↓ Beclin-1	
		↓ Atg5	
		↑ p62	
Rats			
Sprague-Dawley (male, 6–8 weeks old)	High sugar/fat diet + 40 mg/kg STZ (single injection)	↑ LC3-II	Cai et al. (2017)
Sprague-Dawley (male)	STZ injection	↑ Beclin-1	Wang et al. (2017)
		↑ LC3-II	
		↑ Atg12-Atg5	
		↑ Atg3	
Sprague-Dawley (male, 2 months old)	35 mg/kg STZ (single injection)	↓ LC3-II	Shruthi et al. (2017)
Sprague-Dawley (male, 7–8 weeks old)	60 mg/kg STZ (single injection)	↑ Beclin-1	Park et al. (2018)
		↑ LC3-II/LC3-I	
		↑ ph-AMPK	
		↓ ph-mTOR	
Sprague-Dawley (male, 6–8 weeks old)	40 mg/kg STZ (single injection)	↓ LC3-II	Mao et al. (2019)
		↓ LC3-II/LC3-I	
BBZDR/Wor (male, 5 months old)	BBZDR/Wor: type 2 diabetic model	↓ Atg9	Qi et al. (2020)
		↓ Atg7	
		↓ LC3	
		↓ Beclin-1	

(DR: diabetic retinopathy; STZ: streptozotocin).

A very recent study by Madrakhimov and colleagues demonstrated that long-term hyperglycemia causes mTOR inhibition leading to autophagy dysregulation (Madrakhimov et al., 2021). Indeed, inhibition of the mTORC1 pathway in STZ-induced diabetic mice was associated with upregulation of Beclin-1 in the entire inner retina and ATG9A in NeuN (Neuronal Nuclei) positive RGCs. These changes were accompanied with signs of neuronal cell damage, such as activation of cleaved caspase three and decrease of the total number of cells in the GCL. Interestingly, blockade of autophagy by mTOR activator-MHY1485 injections in diabetic mice resulted in a prominent rescue of neuronal cells (Madrakhimov et al., 2021).

Increased LC3-II expression was also reported by Cai and colleagues (2017) in male rats fed with sugar, high fat diet followed by STZ injection; in this model treatment with Glucagon-like peptide-1 (GLP-1), reduced oxidative stress and reverted the upregulation of LC3-II expression (Cai et al., 2017).

At variance with the previous reported results, in the retina of STZ-induced diabetic mice a decrease of Beclin-1, Atg7, p62 and LC3-II expression was reported as compared to control group; treatment with the heparanase inhibitor PG545 promoted autophagy and inhibited the secretion of pro-inflammatory cytokines alleviating diabetic retinopathy (Wang et al., 2020).Similarly, in STZ-induced diabetic C57BL/6J mice, as well as in Bio-Breeding Zucker diabetic (BBZDR/Wor) rats that spontaneously develop a T2D, Qi and colleagues reported a dramatic reduction of Atg7, Atg9, LC3 and Beclin-1 in diabetic retina as compared to controls (Qi et al., 2020).

Interestingly, in this same study the authors showed a diurnal rhythmicity of Atg proteins levels. Under basal conditions Atg9 and LC3 expression showed a biphasic diurnal cycle with two peaks of highest and lowest levels, respectively, while Atg7 and Beclin-1 had a monophasic 24 h cycle. In the retinas from both T1D and T2D mice a significant impairment of Atg proteins diurnal rhythmicity was reported (Qi et al., 2020). This suggests that in diabetic retina the molecular circuit regulating basal autophagy, in terms of intensity and duration, might be altered.

Suppression of LC3-II expression was also reported by Mao and colleagues in STZ-induced diabetic rats; the reduced level of LC3-II correlated with a significant upregulation of a specific microRNA (miRNA), miR-204-5p. Indeed, anti-miR-204-5p treatment enhanced the expression of LC3-II and increased LC3-II/LC3-I ratio, while miR-204-5p mimic treatment was associated with opposite effects thus suggesting that in DR miR-204-5p is responsible for the inhibition of the autophagy pathway (Mao et al., 2019).

The modulation of autophagy in diabetic models may vary depending on the progression of the disease and therefore on the time points analyzed. In RGCs of C57BL/KsJ-db/db mouse, a rodent model of spontaneous diabetes, Fu and co-workers (2020) observed a fluctuating modulation of LC3-II protein levels depending on the age of diabetic mice without identifying a clear trend (Fu et al., 2020). In db/db mice Luo and colleagues reported a downregulation of pro-autophagy proteins like LC3-II, Beclin-1 and Atg5 and a significant upregulation of p62 (Luo et al., 2021). More interestingly, in STZ-induced diabetic rats Shruthi and collaborators (2017) observed a biphasic modulation of LC3-II retinal expression characterized by an increase in 2 months old followed by a significant decrease in 4 months old diabetic rats when compared to control animals. The initial upregulation of the pathway could be part of the adaptive response to the damage induced by hyperglycemia. On the other hand, the later impairment of autophagy might be the consequence of the system overload due to the prolonged diabetic-related damage and contribute to the apoptotic retinal cell death (Shruthi et al., 2017).

A recent study by Park and collaborators (2018) focused on the role of autophagy on RGC survival depending on the type of triggering injury (Park et al., 2018). Autophagy was upregulated in both diabetic and glaucomatous retinas, however while autophagy inhibition, by 3-methyladenine (3-MA), an inhibitor of phosphatidylinositol 3-kinases (PI3K), decreased the apoptosis of RGCs in glaucomatous retina, it failed in rescuing RGCs in diabetic retina. The work by Park and collaborators suggests that, depending on the type of injury and the intracellular pathway engaged for cell death, autophagy could either promote RGC survival or death (Park et al., 2018).

Interestingly in a *drosophila* model of hyperglycemia developed by raising adult fruit flies under high-sucrose regimens, signs of autophagy deregulation, such as significant and progressive increase of LC3 and p62 staining, with accumulation of autophagosomes were observed in eye sections (Catalani et al., 2021).

In murine retinal explant, exposure to HG was associated with reduced LC3-II levels and upregulation of the cargo-protein p62. Treatment with octreotide, an analogue of somatostatin, prevented the autophagy changes induced by HG, and exerted anti-apoptotic effects. Co-treatment with the autophagy inhibitor chloroquine (CQ) reverted the neuroprotective effects of octreotide suggesting that a cross talk between autophagy and apoptosis occurs in the injured retina (Amato et al., 2018).

## The Role of Autophagy in *In Vitro* Models of Diabetic Retinopathy

Retinal lesions observed over the course of DR are initially characterized by pericyte cell death, which generates ischemia and promotes the extravasation of plasma constituents such as low-density lipoproteins (LDLs). This generates the damage of RPE and activation of microglial and Müller cells (Fu et al., 2012). On the other hand, DR induced neuronal dysfunction, with RGCs death, apoptosis of amacrine cells in INL, loss of synapses and dendrites and alteration of synaptic activity (Ozcan et al., 2006; Oshitari et al., 2011). It is clear that a large number of cellular elements in the retina are affected by DR (Yang et al., 2020) and therefore, several *in vitro* studies have focused on the modulation of autophagy in the different cell types exposed to diabetes-related insults (Table 2).

**TABLE 2:** Autophagy modulation in cell culture models of diabetic retinopathy.

Cell culture	DR model	Autophagy markers	References
RPE			
hTERT-RPE (telomerase-immortalized human RPE cells)	HOG-LDL -highly oxidized glycated-LDL	↑ LC3-II	Du et al. (2013)
ARPE-19 (human immortalized RPE cells)	HG: 30 mM, 48 h	↑ LC3-II	Yao et al. (2014)
		↑ autophagosomes	
		↓ p62	
ARPE-19	HG: 30 mM, 48 h	↑ autophagosomes	Shi et al. (2015)
		↑ LC3-II	
hiPSC-RPE (human induced pluripotent stem-cell-derived retinal pigment epithelium cell lines)	HG: 25 mM, 5 weeks	↑ p62	Kiamehr et al. (2019)
Müller cells			
rMC-1 (rat retinal Müller cells)	HG: 25 mM, 24 h	↑ LC3-II	Lopes de Faria et al. (2016)
		↑ Beclin-1	
		↑ p62	
MIO-M1 (immortalized human Müller cell line)	HOG-LDL-highly oxidized glycated-LDL	↑ LC3-II	Fu et al. (2016a)
		↑ Beclin-1	
		↑ Atg5	
rMCs (primary rat Müller cells)	HG: 30–60 mM, 24–48 h	↓ LC3-II	Chen et al. (2018)
		↓ Beclin-1	
rMCs	HG: 40 mM, 24 h	↓ Beclin-1	Wang et al. (2019b)
		↓ LC3-II	
		↑ p62	
Pericytes			
HRCPs (human retinal capillary pericytes)	HOG-LDL-highly oxidized glycated-LDL	↑ LC3-II	Fu et al. (2016b)
		↑ Beclin-1	
		↑ Atg5	

(DR: diabetic retinopathy; LDL: low density lipoprotein; HG: high glucose).

### Retinal Pigment Epithelial Cells

Exposure of human immortalized RPE cell, ARPE-19, to high glucose (HG) induced a significant upregulation of autophagy flux. Compared to cells cultured under normal glucose condition, cultures exposed to HG showed increased autophagosome formation, upregulation and changes in the expression pattern of LC3-II and reduction of p62 levels. HG-induced autophagy was mainly regulated through the ROS-mediated ER stress and independent of mTOR signaling pathway (Yao et al., 2014). Similarly, in the same cell line exposed to HG, Shi and colleagues (2015), showed activation of autophagy by reporting an increase of autophagosome number and upregulation of LC3-II protein expression. Under these experimental conditions, inhibition of autophagy obtained by pre-treatment with 3-MA, induced accumulation of damaged-mitochondria, increased the activity of interleukin-1β (IL-1β) and NLRP3 (a NOD-like receptor family pyrin domain containing three inflammasome responsible for the processing of pro-IL1β to the active form of IL-1β) and reduced cell survival (Shi et al., 2015). Altogether, these experimental observations would suggest that in RPE cells exposed to HG stress induction of autophagy represents a cytoprotective response.

Accordingly, treatment with fenofibrate, a peroxisome proliferator-activated receptor alpha (PPARα) agonist by preventing ER-stress and inducing autophagy, exhibited a protective effect in RPE cells exposed to hyperglycemia (25 mM, 18 days) and hypoxia (1% oxygen, for 6 h or 24 h), two components of the diabetic milieu (Miranda et al., 2012; Lazzara et al., 2020).

More recently, Kiamehr and co-workers (2019) using human induced pluripotent stem-cell-derived retinal pigment epithelium (hiPSC-RPE) cell lines, obtained from T2D and healthy control patients, evaluated the effects of hyperglycemia, in the presence of absence of added insulin, on cellular functionality and autophagy (Kiamehr et al., 2019). The authors did not detect any differences in LC3-II expression between diabetic or healthy control hiPSC-RPEs, whereas they observed a significant p62 accumulation in T2D hiPSC-RPE as compared to healthy control (Kiamehr et al., 2019). This change in p62 expression might be unrelated to the autophagy pathway, since p62 is involved in several other functions; one possible hypothesis is that this upregulation of p62 is linked to the antioxidative NRF-2ARE pathway (nuclear factor erythroid-2 related factor/antioxidant response elements) evoked by the energy depletion in diabetic cells (Jain et al., 2010; Felszeghy et al., 2019).

In addition to hyperglycemia, extravasation of plasma lipoproteins modified by oxidation and glycation are important factors driving DR and leading to cytotoxicity (Yu and Lyons, 2013). In telomerase-immortalized human RPE (hTERT-RPE) cells treated with in vitro-modified highly oxidized glycated- (HOG-) LDL, reduced viability was accompanied by the induction of LC3-II expression with no changes in Beclin-1 protein level (Du et al., 2013). Pre-treatment with either native-High-density lipoprotein (N-HDL) or HOG-HDL inhibited HOG-LDL-induced LC3-II expression and partially mitigated RPE cell death (Du et al., 2013).

### Retinal Müller Cells

Retinal Müller cells (rMCs), the primary retinal glial cells, make contacts with every cell type in the retina and are necessary for both neuronal and vascular function and viability (Shen et al., 2014).

The role of autophagy in modulating rMCs response to HG was investigated by Lopes de Faria and collaborators (Lopes de Faria et al., 2016). The study showed that rMCs exposed to HG upregulated the initial steps of autophagy, as shown by increase of Beclin-1 and LC3-II protein expression levels; however, the process of cargo degradation could not be completed due to the overcome of lysosomal dysfunction. The latter caused accumulation of p62 that, in turn, led to VEGF release and rMCs apoptosis. Inhibition of the initial stage of autophagy with 3-MA or the final stage with Bafilomycin A1 (a vacuolar-type H^+^-ATPase inhibitor) increased the number of apoptotic rMCs under either normal condition or following exposure to diabetic milieu conditions. On the contrary, induction of autophagy by rapamycin, a mTOR inhibitor, upregulated Beclin-1 expression, prevented p62 accumulation by restoring autophagy cargo degradation and protected cells from apoptosis (Lopes de Faria et al., 2016).

Comparable results were reported by Wang and collaborators (2019) in a similar cell culture model of primary rat rMCs. Following exposure of rMCs to HG, the authors detected a downregulation of autophagy with reduction of Beclin-1 and LC3-II expression and accumulation of p62 (L. Wang et al., 2019a). Treatment with epigallocatechin gallate (EGCG), a polyphenol present in green tea, protected cells from apoptosis by activating autophagy and reestablishing cargo degradation (L. Wang et al., 2019a). Accordingly, in rat primary rMCs the number of autophagic/lysosomal vacuoles was reduced after exposure to HG; this observation, together with the reported decrease of LC3-II and Beclin-1 protein expression suggested that autophagy activity in rMCs was inhibited by HG conditions. Under these experimental conditions, treatments with berberine reduced HG-induced rMCs apoptosis at least in part by enhancing autophagy (Chen et al., 2018).

Upregulation of Atg5, Beclin-1 and LC3-II proteins were reported in spontaneously immortalized human Müller cell line (MIO-M1) exposed to in vitro-modified HOG-LDL. Müller cell death was partially prevented by inhibiting autophagy with 3-MA or by knocking down Atg5 and Beclin-1 suggesting that autophagy takes part to the apoptosis induced by HOG-LDL (Fu et al., 2016a).

### Pericytes

Pericyte cell death is one of the early features of DR (Hammes et al., 2002). Fu and colleagues (2012, 2016) investigated the modulation of autophagy in human retinal capillary pericytes (HRCP) exposed to HOG-LDL (Fu et al., 2012; Fu et al., 2016b) showing a significant dose-dependent increase of LC3-II, Atg5 and Beclin-1 (Fu et al., 2016b). In this study, autophagy appeared to play a dual role depending of the HOG-LDL concentrations: exposure to low levels of HOG-LDL was associated with a pro-survival autophagy response, on the contrary, when the cells were exposed to higher HOG-LDL concentration autophagy promoted cell death (Fu et al., 2016b).

## Mitophagy and Diabetic Retinopathy

Mitophagy is a specialized form of autophagy responsible for the quality and quantity control of mitochondria (Pickles et al., 2018). These organelles are the primary source of cellular energy (ATP production), involved in respiration and metabolic processes (Kowluru, 2005) and a key source of ROS in diabetes (Sivitz and Yorek, 2010; Hammes, 2018). Oxidative stress originating in mitochondria from endothelial cell has been reported to alter several independent pathways, each contributing to the development of microvascular complications in DR (Du et al., 2000; Nishikawa et al., 2000). Furthermore, the increase of oxidative stress during hyperglycemia damages itself mitochondria function and structure (Madsen-Bouterse et al., 2010). Indeed, retina of diabetic patients and diabetic rodents showed accumulation of damaged and dysfunctional mitochondria (Masser et al., 2017; Kowluru and Mishra, 2018).

Recently, Zhou and co-workers (2019) showed activation of mitophagy in the retinas of diabetic (db/db) mice (Zhou et al., 2019). Indeed, a significant increase of mitophagy associated protein, PINK-1 and Parkin, was reported in the retinas of db/db mice as compared with non-diabetic (db/m) mice together with the upregulation of LC3-II/LC3-I ratio and reduction of p62. PINK1 (PTEN induces putative kinase protein 1) is a mitochondrially localized serine/threonine protein kinase (Valente et al., 2004) responsible for activation and translocation of Parkin, an E3 ubiquitin-ligase (Kitada et al., 1998), from the cytoplasm to damaged mitochondria (Matsuda et al., 2010). Parkin then marks damaged mitochondria with ubiquitin chains targeting them to mitophagy (Bingol et al., 2014). Accordingly, to the *in vivo* data, rMC-1 cells exposed to HG displayed significant increase of PINK1, Parkin and LC3-II/LC3-I expression as compared to cells exposed to normal glucose (Zhou et al., 2019).

Zhang and collaborators (2019) demonstrated that while the exposure of ARPE-19 cell cultures to low glucose (LG) (15 mM) induced autophagy, treatment with HG (50 mM) was associated with ROS mediated inhibition of mitophagy and reduced proliferative abilities (Zhang et al., 2019). Under HG conditions PINK1 and Parkin were downregulated and exogenous overexpression of these proteins, which reestablished mitophagy, reduced apoptosis and promoted cellular proliferation (Zhang et al., 2019). Intriguingly, the study by Zhang et al. (2019) showed that LG recruit LC3 to mitochondrial fraction suggesting that this condition may specifically induce mitophagy in RPE cells (Zhang et al., 2019).

Devi and colleagues reported induction of mitochondrial damage and mitophagy in rMCs exposed to HG (Devi et al., 2017) that were mediated by the upregulation of thioredoxin-interacting protein (TXNIP), a pro-oxidative stress and pro-apoptotic protein strongly induced by diabetes and HG conditions (Singh, 2013).

In a recent study, Taki and co-workers (2020), using 661W cells, a transformed murine cone cell line, observed that HG treatment (25 mM, 48 h) induced changes in mitophagy and autophagy with mitochondria accumulation and upregulation of p62. Treatment with 3-MA caused a greater increase of p62, superoxides and caspase 3/7 activation suggesting that impairment of the autophagy pathway correlates with superoxide formation and induction of apoptosis (Taki et al., 2020).

In spontaneous Ins2^Akita^ diabetic mouse model, Hombrebueno and colleagues (2019), showed a time-dependent modulation of mitophagy (Hombrebueno et al., 2019). Indeed PINK1-dependent mitophagy in both Müller cells and photoreceptors was exacerbated within the first 2 months of diabetes, while a significant impairment of the pathway was reported in the advanced stages of neurovascular dysfunction (8 months of diabetes). Furthermore, during prolonged diabetes, impairment of mitophagy correlated with the development of premature outer retina senescence (Hombrebueno et al., 2019).

## Concluding Remarks

**FIGURE 2:**
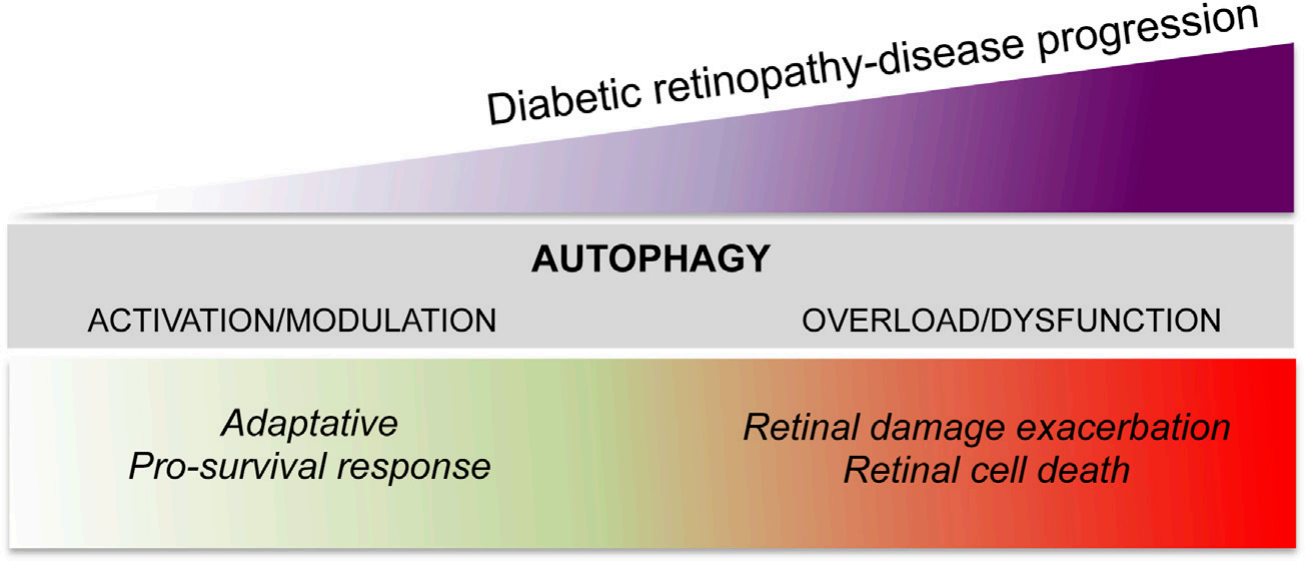
The dual role of autophagy in diabetic retinopathy.

Autophagy in DR has become an area of intense research, however, despite the studies currently available, the question of whether autophagy is counteracting or favoring the evolution of DR remains unclear. Furthermore, controversial results have often been reported in terms of the type of autophagy modulation induced by hyperglycemia (induction *vs* impairment), in both *in vitro* and *in vivo* models (Gong et al., 2021). However, most evidence suggests that autophagy may act with a damage/time-dependent double action (Figure 2). Under mild stress or during the initial phase of DR, autophagy acts as an adaptative response with pro-survival and anti-apoptotic effects (Dehdashtian et al., 2018); on the other hand, under severe stress and in the later phase of DR, dysregulated autophagy, as a consequence of the system overload due to the prolonged damage, contributes to the apoptotic retinal cell death exacerbating the damage (Fu et al., 2016b; Shruthi et al., 2016).

In must also be stressed that the claim of some studies related to the induction or inhibition of autophagy are often not supported by the data. Indeed, being a dynamic process, autophagy should be studied in terms of flux. A simply increased number of autophagosomes, either by LC3 immunofluorescent staining or by transmission electron microscope (TEM), as well as changes in LC3-II/LC3-I ratio detected by western blot are not enough to drawing conclusion on the kind of autophagy activity modulation (Abudu and Acevedo-Arozena, 2021; Klionsky et al., 2021). Therefore, the use of more specific experimental settings, i.e. measurement of autophagosome substrates degradation, comparison of LC3-II accumulation in the absence or presence of lysosomal enzymatic activity inhibitors, should be performed to be able to state the occurrence of an autophagic process (Rubinsztein et al., 2009; Klionsky et al., 2021). It should be also taken into consideration that autophagy activity varies with animal age, sex or strain background and it also undergoes a diurnal rhythmicity. All these factors might affect the final results introducing a complex variability among the different experimental settings and making difficult a direct comparison of the different studies.

Based on the data accumulated so far, interpreting the contribution of autophagy in DR is still difficult and further studies are guaranteed in order to unravel the possibility that pharmacological modulation of the pathway could be exploited for DR supportive therapies.
